# Parental health literacy and advanced bone age among school-aged children with overweight or obesity: a cross-sectional study

**DOI:** 10.3389/fpubh.2026.1946206

**Published:** 2026-10-14

**Authors:** Dan Wang, Simin Wang, Juan Liang, Lu An

**Affiliations:** Department of Pediatrics, Xijing Hospital, the Fourth Military Medical University, Xi'an, China

**Keywords:** age determination by skeleton, health literacy, parents, pediatric nursing, pediatric obesity

## Abstract

**Background:**

Existing studies have shown that parental health literacy is closely associated with the development of childhood overweight and obesity. Childhood overweight and obesity, in turn, can lead to a range of health outcomes, including adult obesity, type 2 diabetes, and advanced bone age. However, no studies have yet examined the relationship between parental health literacy and advanced bone age in overweight and obese children.

**Methods:**

Overweight and obese children were classified into two groups based on whether their bone age was advanced. Baseline characteristics between the two groups were compared. Multivariate logistic regression was used to analyze the association between health literacy and advanced bone age. Multivariate linear regression was performed for sensitivity analyses, with sequential adjustment for age, sex, BMI SDS, and other factors. Receiver operating characteristic (ROC) curve analysis was used to evaluate the discriminative ability of parental health literacy for advanced bone age. Finally, stratified analyses were conducted to assess differences in the strength of the association across subgroups.

**Results:**

Higher levels of parental health literacy were associated with a lower risk of advanced bone age in children. Paternal health literacy was influenced by confounding factors, whereas maternal health literacy showed a more consistent association across the fitted models. However, the formal comparison showed no statistically significant difference between the regression coefficients for maternal and paternal health literacy (*χ*^2^ = 0.444, *p* = 0.505). Exploratory ROC curve analysis showed that adding maternal health literacy to a model based on basic characteristics improved discriminative ability (AUC = 0.734), compared with adding paternal health literacy (AUC = 0.717) or combining both parents’ health literacy (AUC = 0.733). The negative association between maternal health literacy and advanced bone age was more pronounced among mothers with higher educational levels.

**Conclusion:**

These findings suggest that, in pediatric nursing practice, parental health literacy, particularly maternal health literacy, can be improved through health education and other approaches to better support healthy skeletal growth and development in overweight and obese children.

## Introduction

Childhood overweight and obesity have become a major global public health issue, with its prevalence increasing year by year. Studies have shown that in China, the prevalence of overweight and obesity among children increased from 11.7% in 1991 to 25.2% in 2011, with primary school-aged children and boys identified as high-risk groups (1). A study of school-aged children aged 5–12 years in India reported that the prevalence of obesity was 38.4% (2). This further highlights the widespread prevalence of childhood obesity across different countries and regions and its public health significance. Obesity has significant impacts on children’s physical health and growth and development. In terms of metabolism, obesity is closely associated with insulin resistance (3), type 2 diabetes (4), and dyslipidemia. In the cardiovascular system, it can lead to elevated blood pressure and early atherosclerotic changes (5, 6). In the skeletal and musculoskeletal systems, it may result in flat feet (7), genu valgum, abnormal bone mineral density, and advanced bone age. Advanced bone age refers to a rate of skeletal maturation that exceeds chronological age (8), which may lead to reduced adult height and adversely affect physical and mental health (9, 10). Therefore, exploring the factors influencing advanced bone age in overweight and obese children has important clinical significance.

Health literacy is defined as an individual’s ability to understand, access, and use health information to improve their own and others’ well-being. It plays an important role in the management of chronic diseases (11). A review has shown that health literacy is recognized as a fundamental and modifiable factor in the development and maintenance of obesity (12). Low levels of health literacy, including limited ability to read and interpret food labels and estimate portion sizes, can contribute to disordered eating behaviors (DEB) and obesity (13, 14) As children have limited cognitive and information-processing abilities, they rely heavily on parental guidance and support in health-related decision-making. Therefore, parental health literacy is crucial to children’s health outcomes.

Parental health literacy may influence the skeletal growth and development of overweight and obese children through multiple biological and behavioral pathways. Parents with higher health literacy may be more likely to understand health information related to nutrition, physical activity, sleep, and weight management and translate it into daily family health behaviors, such as appropriate dietary management and physical activity, thereby reducing children’s energy intake and body weight. Children’s body weight and metabolism may influence skeletal maturation through multiple biological pathways, including insulin/IGF-1-related signaling (15), leptin, and the hypothalamic–pituitary-gonadal (HPG) axis (16). Therefore, parental health literacy may be indirectly associated with advanced bone age by influencing children’s health behaviors and weight-related metabolic status.

Previous studies have demonstrated that childhood obesity is strongly associated with parental factors (17–19), and many studies have also analyzed factors influencing advanced bone age in overweight and obese children (20, 21). However, few studies have focused on parental health literacy. Therefore, whether parental health literacy influences skeletal maturation in overweight and obese children remains insufficiently explored. This study aims to investigate this association and provide a nursing evidence base for interventions in child growth and development.

## Methods

### Study population

This was a cross-sectional study including children who underwent bone age assessment at our hospital between January 2022 and January 2025.

Inclusion criteria

- School-aged children aged 6–12 years;
- According to the World Health Organization (WHO) Child and Adolescent Growth Standards, children with a BMI SDS ≥ 1 were classified as overweight, and those with a BMI SDS ≥ 2 were classified as obese (22);
- Both parents completed the health literacy questionnaire;
- Left-hand wrist radiography was performed and bone age assessment results were available.

Exclusion criteria

- Other diseases that may affect growth and development, such as clinically diagnosed central precocious puberty (CPP) (23) and growth hormone deficiency;
- Secondary obesity;
- Use of glucocorticoids, growth hormone, or sex hormones within the past three months;
- End-stage malignancy or severe systemic diseases such as severe hepatic or renal insufficiency;
- Missing key data.

Both parental health literacy assessments and the child’s bone-age assessment were completed during the same outpatient visit. Parents completed the HLS19-Q12 questionnaire before receiving the child’s bone-age assessment results. The detailed participant inclusion and exclusion process is shown in Supplementary Figure 1.

### Quality control

All imaging data were independently reviewed by two qualified pediatric radiologists who were blinded to clinical information. Skeletal maturation stages were assessed, and each bone’s maturity score was calculated according to the TW3 (Tanner–Whitehouse 3) bone age assessment system. The scores of individual bones were summed to obtain a total score, which was then converted into bone age. In cases of disagreement, consensus was reached through discussion. The intraclass correlation coefficient (ICC) was calculated to assess inter-rater agreement. The agreement between the two radiologists was excellent (ICC = 0.949, 95% CI: 0.923–0.971). Missing data for “residence” were observed in 15 participants (7.14%) and were handled using multiple imputation by chained equations (MICE), and 20 imputed datasets were generated. Regression analyses were performed separately in each imputed dataset, and the estimates from the imputed datasets were pooled according to Rubin’s rules using the “pool” function in the R mice package. All other variables included in the analyses had no missing data.

### Definition and calculation of key variables

Residential areas were classified according to the urban–rural classification codes issued by the National Bureau of Statistics of China based on the participants’ residential addresses. The 15th digit of the classification code indicates the category, with “1” representing urban residence and “2” representing rural residence. Children’s BMI was calculated as weight (kg) divided by height squared (m^2^). BMI SDS was calculated using the *childsds* package in R software by inputting the child’s age, sex, and BMI. Based on previous literature, advanced bone age was defined as the difference between bone age (BA) and chronological age (CA). When BA − CA > 1, it was classified as advanced bone age; otherwise, it was classified as non-advanced bone age (24).

### Study instruments

This study used the 12-item Health Literacy Survey Questionnaire (HLS_19_-Q12) to assess parental health literacy. The questionnaire was developed by the WHO Health Literacy Measurement Action Network (M-POHL) and has been applied in China (25). The HLS_19_-Q12 consists of a 12-cell matrix (three health domains × four health management tasks). Respondents rate the perceived difficulty of each item using a 4-point Likert scale (1 = very difficult, 4 = very easy). A valid response rate of ≥80% was required for inclusion. The final scores were categorized into four levels: excellent, sufficient, problematic, and inadequate. In this study, health literacy was included as a categorical variable in the analysis, with the final results classified into four levels: Excellent, Sufficient, Problematic, and Inadequate (Supplementary Table 1). Excellent, Sufficient, Problematic, and Inadequate health literacy were coded as 3, 2, 1, and 0, respectively, with higher values indicating higher health literacy. The Cronbach’s α coefficients of the HLS_19_-Q12 for fathers and mothers were 0.800 and 0.812, respectively, indicating good internal consistency.

### Covariate selection

We selected age, sex, BMI SDS, Height SDS, Tanner stage, parental age, parental educational level, and residence as covariates. Specifically, age, sex, BMI, and height are important determinants of skeletal maturation and pubertal development in children and are closely related to bone age development. Parental age and parental educational level are associated with parents’ ability to obtain health information and their health literacy, while residence mainly reflects differences in the socioeconomic and healthcare resource environment of the family and may be associated with parental health literacy and children’s health status. Tanner stage is closely related to pubertal development and skeletal maturation. All of these factors may influence the association between parental health literacy and children’s bone age development; therefore, they were prespecified as potential confounders and included in the multivariable models for adjustment.

### Statistical analysis

Statistical analyses were performed using R software (version 4.4.1). After testing for normality, continuous variables with a normal distribution were presented as mean ± standard deviation, and comparisons between groups were performed using independent-samples *t*-tests. Non-normally distributed variables were expressed as median (interquartile range), and group comparisons were conducted using the Mann–Whitney U test. Categorical variables were analyzed using the chi-square test or Fisher’s exact test when expected frequencies were small, and are presented as counts and percentages [*n* (%)]. Multivariate logistic regression analysis was conducted with the presence of advanced bone age as the dependent variable. Tanner stages I–III were coded as 0–2, while parental education levels of “Junior high school or below,” “High school/technical secondary school,” and “College degree or above” were coded as 0, 1, and 2, respectively. First, only paternal/maternal health literacy was included. Then BMI SDS was adjusted. Subsequently, age, sex, and BMI SDS were adjusted. Finally, all baseline characteristics (age, sex, BMI SDS, Height SDS, Tanner stage, parental age, parental educational level, and residence) were adjusted to evaluate the association between parental health literacy and advanced bone age. Multiple linear regression analysis was performed using bone age difference (BA−CA) as a continuous outcome variable, with the same adjustment strategy to verify the robustness of the primary findings. Receiver operating characteristic (ROC) curve analysis was used to assess the discriminative ability of models including: (1) baseline characteristics only (ROC-1); (2) baseline characteristics plus maternal or paternal health literacy (ROC-2; ROC-3); and (3) baseline characteristics plus both paternal and maternal health literacy (ROC-4). The optimal cutoff value for the ROC analysis was determined by maximizing the Youden index. All statistical tests were two-sided, and a *p* value < 0.05 was considered statistically significant. Interaction analyses were performed using multivariable logistic regression models. The models included the main effects of parental health literacy and maternal educational level, as well as their product term. To balance confounding control and model complexity, the interaction models were additionally adjusted for age, sex, and BMI SDS. Parental health literacy was coded from 0 to 3, and maternal educational level was coded from 0 to 2 and treated as an ordinal variable. The statistical significance of the interaction-term coefficient was assessed using the Wald test. Because maternal educational level was modeled as an ordinal variable, the interaction term represented a 1-degree-of-freedom test for a linear interaction trend rather than a global test of interaction across educational categories. To assess the robustness of the assumption that the association with advanced bone age is relatively consistent for each one-level increase in parental health literacy when health literacy was coded as an ordinal variable from 0 to 3, we additionally performed a sensitivity analysis by treating parental health literacy as a categorical variable, with Inadequate as the reference category.

### *Post hoc* power analysis

A *post hoc* power analysis was performed based on the available sample of 210 participants and was used to describe the statistical power of the univariable analyses. For continuous variables, the statistical power was 0.946, assuming a medium effect size (Cohen’s *d* = 0.50) and a two-sided significance level of 0.05. For categorical variables, assuming a medium effect size (Cohen’s *w* = 0.30) and a significance level of 0.05, the statistical power was 0.992 for 2 × 2 contingency tables and 0.980 for 2 × 3 contingency tables. These results indicated that the present study had adequate statistical power with the available sample size.

## Results

### Baseline characteristics of overweight and obese children and their parents in the non-advanced and advanced bone age groups

A total of 210 children were included in the study, among whom 90 (42.9%) were identified as having advanced bone age. The results showed that multiple indicators differed significantly between the advanced bone age group and the non-advanced group, including BMI SDS, Tanner stage, age, and maternal education level (*p* < 0.05). Specifically, BMI SDS and Tanner stage were higher in the advanced bone age group, whereas age and maternal education level were lower compared with the non-advanced group (Table 1). We also compared the baseline characteristics between participants included in the study and those excluded due to missing key data. The results showed that there were no statistically significant differences in the main baseline characteristics between the two groups (Supplementary Table 2). Among the 210 fathers, 12 (5.7%) were classified as having Inadequate health literacy, 38 (18.1%) as Problematic, 99 (47.1%) as Sufficient, and 61 (29.0%) as Excellent. Among the 210 mothers, 7 (3.3%) were classified as having Inadequate health literacy, 36 (17.1%) as Problematic, 90 (42.9%) as Sufficient, and 77 (36.7%) as Excellent.

**Table 1:** Comparison of the basic characteristics of obese children with non-advanced and advanced bone age and their parents.

Characteristics	Non-advanced group (*n* = 120)	Advanced group (*n* = 90)	*p* value
BMI SDS	2.3 (2.0–2.6)	2.4 (2.1–2.7)	0.030
Age (years)	9.4 (8.6–10.0)	8.8 (8.2–9.7)	0.013
Sex			0.531
Male	73 (60.83%)	50 (55.56%)	
Female	47 (39.17%)	40 (44.44%)	
Height SDS	0.47 ± 0.66	0.49 ± 0.76	0.816
Tanner stage			0.044
I	73 (60.83%)	44 (48.89%)	
II	40 (33.33%)	32 (35.56%)	
III	7 (5.83%)	14 (15.56%)	
Fathers’ age (years)	39 (38–41)	39 (37–40)	0.306
Mothers’ age (years)	37 (35–39)	37 (35–38)	0.601
Fathers’ educational level			0.093
Junior high school or below	13 (10.83%)	10 (11.11%)	
High school/technical secondary school	61 (50.83%)	58 (64.44%)	
College degree or above	46 (38.33%)	22 (24.44%)	
Mothers’ educational level			0.003
Junior high school or below	8 (6.67%)	8 (8.89%)	
High school/technical secondary school	77 (64.17%)	73 (81.11%)	
College degree or above	35 (29.17%)	9 (10%)	
Residence			0.789
Urban	93 (77.5%)	72 (80%)	
Rural	27 (22.5%)	18 (20%)	

Continuous variables with a normal distribution are presented as mean ± standard deviation (SD) and were compared using independent-samples *t*-tests. Non-normally distributed continuous variables are presented as median (interquartile range, IQR) and were compared using the Mann–Whitney *U* test. Categorical variables are presented as counts and percentages [*n* (%)] and were compared using the chi-square test or Fisher’s exact test when appropriate.

### Association between paternal health literacy and advanced bone age

Multicollinearity analysis showed that the variance inflation factors (VIFs) for all variables were slightly above 1 and well below 5, indicating no evidence of multicollinearity (Supplementary Table 3). In the model including only paternal health literacy, paternal health literacy was significantly negatively associated with advanced bone age (OR = 0.912, *p* = 0.024). After adjustment for BMI SDS, this negative association remained significant (OR = 0.920, *p* = 0.039). After further adjustment for age and sex, the negative association also remained significant (OR = 0.916, *p* = 0.030). However, after adjusting for all baseline characteristics, the association between paternal health literacy and advanced bone age was no longer statistically significant (OR = 0.930, *p* = 0.073). These results indicate that for each one-level increase in paternal health literacy, the odds of advanced bone age in children decreased by 7.0–8.8%. Multiple linear regression analysis further indicated a negative association between paternal health literacy and the outcome (*β* = −0.180 to −0.159, *p* = 0.046–0.085), although statistical significance was not consistent. After stepwise adjustment for confounding factors, this association was no longer significant (Table 2).

**Table 2:** Association between fathers’ health literacy and advanced bone age.

Model	Term	Multivariable logistic regression				Multiple linear regression	
		*β*	*p* value	OR	95%CI	*β*	*p* value
Model 1	Fathers’ health literacy	−0.092	0.024	0.912	0.842–0.987	−0.180	0.046
Model 2	Fathers’ health literacy	−0.084	0.039	0.920	0.850–0.995	−0.167	0.064
	BMI SDS	0.160	0.034	1.174	1.013–1.360	0.254	0.130
Model 3	Fathers’ health literacy	−0.087	0.030	0.916	0.847–0.991	−0.173	0.055
	BMI SDS	0.164	0.029	1.178	1.018–1.363	0.257	0.124
	Age	−0.072	0.018	0.931	0.878–0.987	−0.137	0.043
	Sex	−0.052	0.445	0.949	0.831–1.085	−0.047	0.755
Model 4	Fathers’ health literacy	−0.073	0.073	0.930	0.859–1.006	−0.159	0.085
	BMI SDS	0.141	0.058	1.152	0.996–1.332	0.203	0.227
	Age	−0.058	0.056	0.944	0.889–1.001	−0.122	0.076
	Sex	−0.056	0.411	0.946	0.828–1.080	−0.056	0.714
	Height SDS	0.049	0.303	1.050	0.957–1.153	0.144	0.184
	Tanner	0.088	0.082	1.092	0.990–1.204	0.139	0.224
	Fathers’ age	−0.019	0.183	0.981	0.954–1.009	−0.055	0.094
	Mothers’ age	−0.006	0.652	0.994	0.967–1.021	−0.003	0.926
	Fathers educational	−0.076	0.161	0.927	0.834–1.030	−0.144	0.240
	Mothers educational	−0.170	0.010	0.844	0.742–0.960	−0.288	0.054
	Residence	0.040	0.621	1.041	0.888–1.220	−0.033	0.858

### Association between maternal health literacy and advanced bone age

Similar to the analysis of paternal health literacy, in the model including only maternal health literacy, maternal health literacy was significantly negatively associated with advanced bone age (OR = 0.893, *p* = 0.007). After adjustment for BMI SDS, this negative association remained statistically significant (OR = 0.902, *p* = 0.014). After further adjustment for age and sex, the association remained significant (OR = 0.907, *p* = 0.020). Unlike paternal health literacy, after adjustment for all baseline characteristics, the negative association remained statistically significant (OR = 0.899, *p* = 0.010). These results indicate that for each one-level increase in maternal health literacy, the odds of advanced bone age in children decreased by 9.3–10.7%. Multiple linear regression analysis further supported these findings, showing a consistent negative association between maternal health literacy and the outcome (*β* = −0.254 to −0.247, *p* = 0.006–0.008), with statistical significance maintained across all models with different covariate adjustments (Table 3).

**Table 3:** Association between mothers’ health literacy and advanced bone age.

Model	Term	Multivariable logistic regression				Multiple linear regression	
		*β*	*p* value	OR	95%CI	*β*	*p* value
Model 1	Mothers’ health literacy	−0.113	0.007	0.893	0.823–0.969	−0.254	0.006
Model 2	Mothers’ health literacy	−0.103	0.014	0.902	0.831–0.979	−0.239	0.011
	BMI SDS	0.154	0.041	1.166	1.007–1.351	0.235	0.160
Model 3	Mothers’ health literacy	−0.097	0.020	0.907	0.837–0.984	−0.230	0.014
	BMI SDS	0.158	0.035	1.171	1.012–1.355	0.238	0.153
	Age	−0.067	0.027	0.935	0.882–0.992	−0.127	0.060
	Sex	−0.039	0.571	0.962	0.842–1.099	−0.017	0.911
Model 4	Mothers’ health literacy	−0.106	0.010	0.899	0.830–0.974	−0.247	0.008
	BMI SDS	0.130	0.080	1.139	0.985–1.316	0.174	0.296
	Age	−0.051	0.087	0.950	0.896–1.007	−0.107	0.115
	Sex	−0.044	0.514	0.957	0.839–1.092	−0.029	0.850
	Height SDS	0.026	0.582	1.026	0.935–1.126	0.092	0.393
	Tanner	0.085	0.087	1.089	0.988–1.201	0.133	0.239
	Fathers’ age	−0.018	0.202	0.982	0.955–1.010	−0.053	0.101
	Mothers’ age	−0.004	0.778	0.996	0.969–1.024	0.002	0.937
	Fathers educational	−0.090	0.096	0.914	0.823–1.016	−0.176	0.149
	Mothers educational	−0.199	0.002	0.820	0.723–0.929	−0.352	0.016
	Residence	0.047	0.560	1.048	0.895–1.227	−0.017	0.926

### Sensitivity analysis

Because Tanner stage is closely related to skeletal maturation and may lie on the causal pathway through which parental health literacy influences skeletal development in children, we further conducted a sensitivity analysis excluding Tanner stage. The results showed that after removing Tanner stage, the OR for maternal health literacy changed from 0.899 to 0.897, while that for paternal health literacy changed from 0.930 to 0.928, with only minimal changes in the effect estimates (Supplementary Table 4). These findings suggest that Tanner stage did not materially affect the association between parental health literacy and advanced bone age. When parental health literacy was entered into the multivariable logistic regression model as a categorical variable, the ORs for advanced bone age for fathers in the Problematic, Sufficient, and Excellent groups compared with the Inadequate group were 0.658 (*p* = 0.009), 0.588 (*p* < 0.001), and 0.631 (*p* = 0.002), respectively. For maternal health literacy, the corresponding ORs were 0.950 (*p* = 0.794), 0.705 (*p* = 0.061), and 0.688 (*p* = 0.046), respectively (Supplementary Table 5). Overall, higher levels of health literacy were associated with lower odds of advanced bone age, and the direction of the associations was generally consistent with that observed in the ordinal analysis.

### Joint analysis of parental health literacy

To compare whether the associations of parental health literacy with advanced bone age differed significantly, maternal and paternal health literacy were simultaneously included in the multivariable logistic regression model. Maternal health literacy was significantly negatively associated with advanced bone age (OR = 0.911, 95% CI: 0.838–0.991, *p* = 0.031), whereas paternal health literacy was not significantly associated with advanced bone age (OR = 0.953, 95% CI: 0.878–1.035, *p* = 0.254) (Supplementary Table 6). However, a formal comparison of the two regression coefficients using a linear hypothesis test showed no statistically significant difference between them (*χ*^2^ = 0.444, *p* = 0.505).

### ROC curve analysis of the discriminative ability of paternal and maternal health literacy for advanced bone age

In the ROC-1 model including only baseline characteristics, the AUC was 0.711, with a sensitivity of 0.800 and specificity of 0.550. After adding paternal health literacy (ROC-2), the AUC increased to 0.717, while sensitivity decreased to 0.711 and specificity increased to 0.642. After adding maternal health literacy to the baseline model (ROC-3), the AUC increased to 0.734, sensitivity increased to 0.867, and specificity decreased to 0.533. In the model including both paternal and maternal health literacy (ROC-4), the AUC was 0.733, with sensitivity of 0.833 and specificity of 0.550, which was similar to ROC-3 (Table 4; Figures 1A–D). DeLong’s test showed that the difference in AUC between ROC-3 and ROC-1 was statistically significant (*p* = 0.012), whereas no statistically significant differences were observed between ROC-2 and ROC-1 or between ROC-4 and ROC-1. These exploratory ROC results indicate that adding maternal health literacy contributed most to improving model AUC, while combining both parents’ health literacy did not further improve discriminatory performance.

**Table 4:** ROC parameters.

ROC item	AUC	95%AUC	Best-threshold	Youden	Sensitivity	Specificity
ROC-1	0.711	0.642–0.780	0.383	0.350	0.800	0.550
ROC-2	0.717	0.648–0.786	0.415	0.353	0.711	0.642
ROC-3	0.734	0.667–0.800	0.342	0.400	0.867	0.533
ROC-4	0.733	0.666–0.800	0.357	0.383	0.833	0.550

Footnote: ROC-1: Baseline characteristics. ROC-2: Baseline characteristics plus paternal health literacy. ROC-3: Baseline characteristics plus maternal health literacy. ROC-4: Baseline characteristics plus both paternal and maternal health literacy.

**Figure 1:**
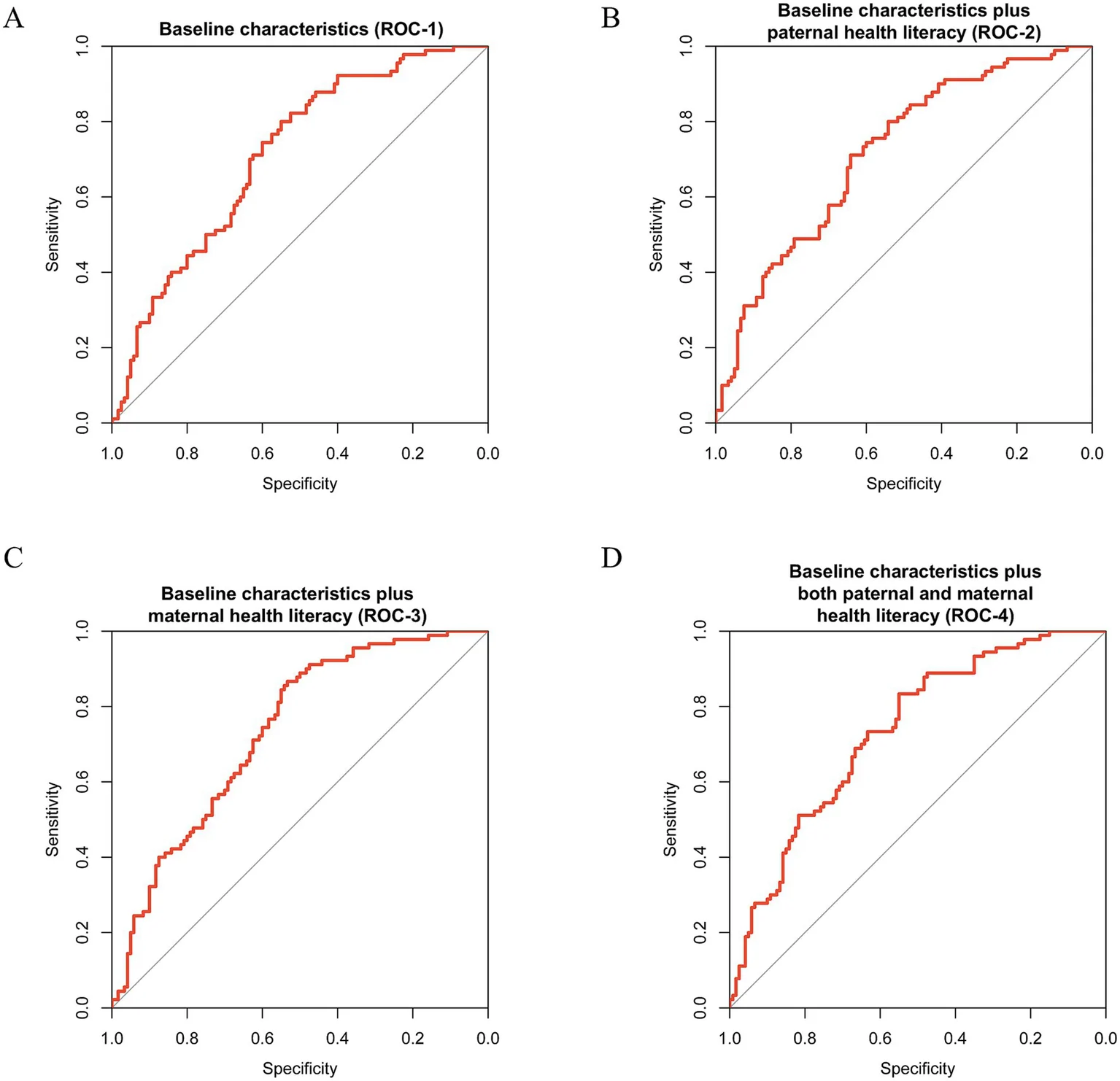
ROC curves for models with different combinations of parental health literacy and baseline characteristics. **(A)** Baseline characteristics (ROC-1). **(B)** Baseline characteristics plus paternal health literacy (ROC-2). **(C)** Baseline characteristics plus maternal health literacy (ROC-3). **(D)** Baseline characteristics plus both paternal and maternal health literacy (ROC-4).

### Stratified analysis

The above analyses showed that maternal educational level differed most markedly between the advanced bone age group and the non-advanced bone age group (*p* = 0.003). To further explore whether maternal educational level influenced the association between maternal health literacy and advanced bone age, we conducted stratified analyses according to maternal educational level. The results showed that when maternal education level was junior high school or below, or high school/secondary vocational school, neither maternal nor paternal health literacy was significantly associated with advanced bone age. However, when maternal education level was college or above, maternal health literacy was significantly negatively associated with advanced bone age (OR = 0.833, *p* = 0.007), whereas paternal health literacy did not reach statistical significance. Interaction analyses showed that the interaction between maternal educational level and maternal health literacy was statistically significant (*P* for interaction = 0.047), whereas the interaction between maternal educational level and paternal health literacy was not significant (*P* for interaction = 0.885) (Table 5; Figure 2).

**Table 5:** Stratified analysis by maternal educational level.

Subgroup	Term	*β*	*p* value	OR	95%CI	*P* for interaction
Junior high school or below	Mothers’ health literacy	−0.103	0.642	0.902	0.600–1.360	0.047
	Fathers’ health literacy	−0.647	0.128	0.524	0.261–1.050	0.885
High school/technical secondary school	Mothers’ health literacy	−0.079	0.120	0.924	0.837–1.020	
	Fathers’ health literacy	−0.032	0.486	0.968	0.885–1.060	
College degree or above	Mothers’ health literacy	−0.183	0.007	0.833	0.735–0.944	
	Fathers’ health literacy	−0.131	0.179	0.878	0.728–1.057	

**Figure 2:**
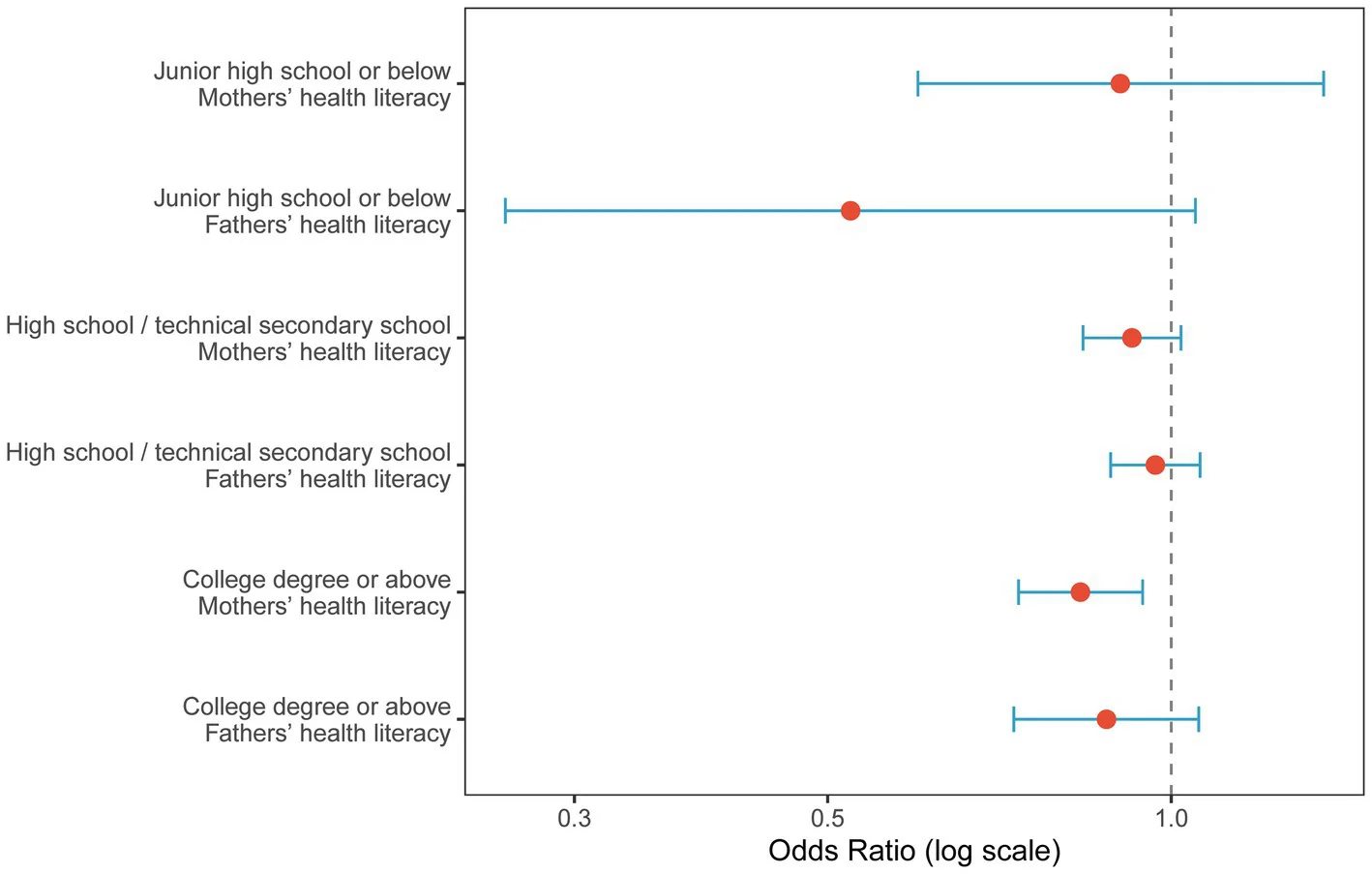
Stratified odds ratios for the association between parental health literacy and advanced bone age by maternal educational level.

## Discussion

This study found that BMI SDS was higher in the advanced bone age group, but the underlying mechanism is complex. The prevailing view suggests that the IGF-1/insulin pathway plays an important role. A classic animal study demonstrated that IGF-1 is not an “auxiliary factor” but a core hormone for skeletal growth with a threshold-dependent effect (26). In overweight and obese children, increased free IGF-1 levels and enhanced IGF-1 signaling promote skeletal growth and development (27). In addition, some studies have suggested that the adrenal axis may act as an early driving factor. For example, Cornelis J. de Groot et al. showed that dehydroepiandrosterone sulfate (DHEAS) is significantly positively correlated with bone age SDS, whereas insulin shows no significant association with bone age (20). The higher Tanner stage in the advanced bone age group is mainly due to premature activation of the hypothalamic–pituitary–gonadal (HPG) axis. Overweight and obese children have higher leptin levels (28), and leptin can directly act on the HPG axis, promoting its early activation, thereby leading to earlier pubertal onset and increased sex hormone secretion.

After separate analyses of paternal and maternal health literacy, we found that paternal health literacy was significantly negatively associated with advanced bone age in unadjusted or minimally adjusted models, whereas this association was no longer significant after adjustment for additional factors, suggesting that the relationship may be influenced by confounding factors. Indicating the need to consider more comprehensive family- and individual-level factors when interpreting the role of paternal health literacy. The association between maternal health literacy and advanced bone age was more consistent, showing a significant negative association regardless of whether baseline characteristics were adjusted for. However, the formal comparison of the maternal and paternal health literacy regression coefficients in the joint model showed no statistically significant difference between them. Therefore, maternal health literacy cannot be considered to have a stronger or more direct association with advanced bone age than paternal health literacy. The ROC curve analysis also supported this finding, with the most notable increase in AUC observed after adding maternal health literacy to the model. However, these models were not subjected to internal validation or calibration assessment and should therefore be considered exploratory. The more consistent association between maternal health literacy and advanced bone age does not imply that mothers play a more central role than fathers in children’s health behaviors. Other factors may also contribute to this finding, such as mothers’ involvement in children’s routine healthcare, socioeconomic factors, and differences in the measurement of parental health literacy.

Stratified analysis revealed that maternal educational level moderated the association between health literacy and advanced bone age. The association between maternal health literacy and advanced bone age was more pronounced among mothers with higher educational levels. This suggests that higher education may enhance the ability to translate health literacy into actual health-related behaviors. In contrast, among mothers with lower educational levels, this association may be attenuated due to limited capacity to understand health information and to implement appropriate health behaviors. Given the relatively small sample size within some educational strata, this finding should be regarded as exploratory and interpreted with caution.

These findings have important implications for nursing practice. Maternal health literacy may represent an important factor associated with advanced bone age in children. Future studies could further investigate the relationship between mother-centered health education, nutritional management, obesity prevention, and skeletal maturation in children.

This study also has several limitations. First, as a cross-sectional study, causal inferences cannot be established, and only associations can be identified. All participants were recruited from a university-affiliated hospital, which may differ from the general community population. Second, other relevant confounding factors, including dietary habits, physical activity, household income, duration and severity of obesity, insulin resistance, growth velocity, and pubertal history, were not fully considered. These factors may be associated with both parental health literacy and skeletal maturation in children; therefore, the associations observed in this study should be interpreted with caution. Third, health literacy was assessed using a questionnaire, which may introduce self-report bias. Urban–rural residence cannot fully capture household socioeconomic differences. In this study, it should be considered as a comprehensive indicator of the social environmental context. Although we conducted a sensitivity analysis excluding Tanner stage, the possibility of overadjustment cannot be completely ruled out. The number of outcome events was relatively limited compared with the number of independent variables, which may have increased the risk of model overfitting and reduced the stability of the effect estimates. Therefore, the results of this study should be interpreted with caution. Future studies should adopt a longitudinal study design to further clarify the role of Tanner stage in the association between parental health literacy and advanced bone age.

## Conclusion

The association between paternal health literacy and advanced bone age varied across the different adjustment models, showing less consistency. The negative association between maternal health literacy and advanced bone age was more consistent across the fitted models and was more pronounced among mothers with higher educational levels. However, the formal comparison of the maternal and paternal health literacy regression coefficients showed no statistically significant difference between their effect estimates (*χ*^2^ = 0.444, *p* = 0.505).

## Data Availability

The raw data supporting the conclusions of this article will be made available by the authors, without undue reservation.
