# Supplementary material for: Parental health literacy and advanced bone age among school-aged children with overweight or obesity: a cross-sectional study
Source: Front Public Health. 2026 Oct 14;14:1946206. doi: 10.3389/fpubh.2026.1946206 (PMC13713633; doi:10.3389/fpubh.2026.1946206)
Supplement: Supplementary file 1 [file Table_1.docx]

Supplementary Table 1. Classification criteria for the four health literacy levels

| Health literacy level | Classification criteria |
| --- | --- |
| Excellent | The proportion of responses rated “very easy” is ≥50%, and the combined proportion of responses rated “very difficult” and “difficult” is <8.334%. In addition, the number of “very easy” responses is >1/2 of all valid items, and the number of “very difficult” + “difficult” responses is ≤1/12 of all valid items. |
| Sufficient | The combined proportion of responses rated “very easy” and “easy” is >83.33%. At least 10 of the 12 items are rated “very easy” or “easy,” and no more than 2 items are rated “very difficult” or “difficult.” |
| Problematic | All respondents who do not meet the criteria for Excellent, Sufficient, or Inadequate health literacy. |
| Inadequate | The proportion of responses rated “very easy” is <8.334%, and the combined proportion of responses rated “very difficult” and “difficult” is ≥50%. In addition, the number of “very difficult” + “difficult” responses is >1/2 of all valid items, and the number of “very easy” responses is ≤1/12 of all valid items. |

| Supplementary Table. 2 Differences in baseline characteristics between the included group and the excluded group with missing key data | | | |
| --- | --- | --- | --- |
|  | Included group (n=210) | Excluded group (n=48) | P value |
| BMI SDS | 2.4 (2-2.7) | 2.4 (2-2.9) | 0.336 |
| Age (years) | 9.1 (8.3-9.9) | 9.1 (8.7-9.7) | 0.736 |
| Sex |  |  | 0.146 |
| male | 123 (58.57%) | 22 (45.83%) |  |
| female | 87 (41.43%) | 26 (54.17%) |  |
| Height SDS | 0.48 ±0.7 | 0.45 ±0.69 | 0.795 |
| Tanner stage |  |  | 0.108 |
| I | 117 (55.71%) | 19 (39.58%) |  |
| II | 72 (34.29%) | 21 (43.75%) |  |
| III | 21 (10.00%) | 8 (16.67%) |  |
| Fathers’ age (years) | 39 (37-40) | 39 (38-40) | 0.824 |
| Mothers’ age (years) | 37 (35-38) | 37 (37-38) | 0.538 |
| Fathers’ educational level |  |  | 0.090 |
| Junior high school or below | 23 (10.95%) | 1 (2.08%) |  |
| High school / technical secondary school | 119 (56.67%) | 26 (54.17%) |  |
| College degree or above | 68 (32.38%) | 21 (43.75%) |  |
| Mothers’ educational level |  |  | 0.137 |
| Junior high school or below | 16 (7.62%) | 0 (0.00%) |  |
| High school / technical secondary school | 150 (71.43%) | 38 (79.17%) |  |
| College degree or above | 44 (20.95%) | 10 (20.83%) |  |
| Residence |  |  | 0.184 |
| Urban | 165 (78.57%) | 33 (68.75%) |  |
| Rural | 45 (21.43%) | 15 (31.25%) |  |

| Supplementary Table 3 Multicollinearity analysis | | | |
| --- | --- | --- | --- |
| Variable | Variance Inflation Factor | Variable | Variance Inflation Factor |
| Fathers’ health literacy | 1.080 | Mothers’ health literacy | 1.052 |
| BMI SDS | 1.043 | BMI SDS | 1.051 |
| Age | 1.052 | Age | 1.052 |
| Sex | 1.044 | Sex | 1.048 |
| Height SDS | 1.055 | Height SDS | 1.057 |
| Tanner | 1.058 | Tanner | 1.058 |
| Father age | 1.054 | Father age | 1.043 |
| Mother age | 1.045 | Mother age | 1.045 |
| Fathers' educational level | 1.056 | Fathers' educational level | 1.063 |
| Mothers' educational level | 1.085 | Mothers' educational level | 1.049 |
| Residence | 1.036 | Residence | 1.038 |

| Supplementary Table. 4 Sensitivity analysis excluding Tanner stage | | | | | | | | |
| --- | --- | --- | --- | --- | --- | --- | --- | --- |
| Covariates | Fathers’ health literacy | | | | Mothers’ health literacy | | | |
|  | β | P value | OR | 95%CI | β | P value | OR | 95%CI |
|  | -0.075 | 0.068 | 0.928 | 0.857-1.005 | -0.109 | 0.009 | 0.897 | 0.827-0.972 |
|  |  |  |  |  |  |  |  |  |
| BMI SDS | 0.145 | 0.053 | 1.156 | 0.999-1.337 | 0.133 | 0.075 | 1.142 | 0.988-1.320 |
| Age | -0.064 | 0.035 | 0.938 | 0.884-0.995 | -0.057 | 0.058 | 0.945 | 0.891-1.002 |
| Sex | -0.054 | 0.429 | 0.948 | 0.829-1.083 | -0.042 | 0.537 | 0.959 | 0.840-1.095 |
| Height SDS | 0.047 | 0.332 | 1.048 | 0.954-1.151 | 0.023 | 0.629 | 1.023 | 0.932-1.123 |
| Father age | -0.017 | 0.232 | 0.983 | 0.956-1.011 | -0.016 | 0.255 | 0.984 | 0.957-1.012 |
| Mother age | -0.009 | 0.514 | 0.991 | 0.964-1.019 | -0.007 | 0.635 | 0.993 | 0.967-1.021 |
| Fathers educational | -0.075 | 0.168 | 0.928 | 0.834-1.032 | -0.089 | 0.099 | 0.915 | 0.823-1.016 |
| Mothers educational | -0.181 | 0.006 | 0.834 | 0.733-0.949 | -0.211 | 0.001 | 0.810 | 0.715-0.918 |
| Residence | 0.052 | 0.520 | 1.054 | 0.899-1.235 | 0.059 | 0.465 | 1.061 | 0.906-1.242 |

| Supplementary Table. 5 Sensitivity analysis treating health literacy as a categorical variable | | | | |
| --- | --- | --- | --- | --- |
| term | β | p value | OR | 95%CI |
| Fathers’ health literacy |  |  |  |  |
| BMI SDS | 0.177 | 0.017 | 1.193 | 1.033-1.378 |
| Age | -0.062 | 0.040 | 0.940 | 0.887-0.997 |
| Sex | -0.030 | 0.653 | 0.970 | 0.850-1.107 |
| Problematic vs Inadequate | -0.419 | 0.009 | 0.658 | 0.482-0.898 |
| Sufficient vs Inadequate | -0.532 | 0.000 | 0.587 | 0.441-0.782 |
| Excellent vs Inadequate | -0.460 | 0.002 | 0.631 | 0.470-0.847 |
| Mothers’ health literacy |  |  |  |  |
| BMI SDS | 0.166 | 0.023 | 1.181 | 1.024-1.361 |
| Age | -0.062 | 0.039 | 0.940 | 0.886-0.996 |
| Sex | -0.046 | 0.493 | 0.955 | 0.836-1.090 |
| Problematic vs Inadequate | -0.051 | 0.794 | 0.950 | 0.648-1.393 |
| Sufficient vs Inadequate | -0.349 | 0.061 | 0.705 | 0.490-1.015 |
| Excellent vs Inadequate | -0.374 | 0.046 | 0.688 | 0.477-0.991 |

| Supplementary Table. 6 Joint Analysis of Parental Health Literacy | | | | |
| --- | --- | --- | --- | --- |
| Term | β | p value | OR | 95%CI |
| BMI SDS | 0.124 | 0.094 | 1.132 | 0.980-1.309 |
| Age | -0.054 | 0.075 | 0.948 | 0.894-1.005 |
| Sex | -0.045 | 0.500 | 0.956 | 0.838-1.090 |
| Height SDS | 0.034 | 0.479 | 1.034 | 0.942-1.136 |
| Tanner | 0.085 | 0.090 | 1.088 | 0.987-1.200 |
| Father age | -0.020 | 0.165 | 0.980 | 0.953-1.008 |
| Mother age | -0.005 | 0.731 | 0.995 | 0.968-1.023 |
| Fathers educational | -0.087 | 0.107 | 0.917 | 0.825-1.019 |
| Mothers educational | -0.183 | 0.006 | 0.832 | 0.732-0.946 |
| Residence | 0.046 | 0.565 | 1.047 | 0.895-1.226 |
| Fathers’ health literacy | -0.048 | 0.254 | 0.953 | 0.878-1.035 |
| Mothers’ health literacy | -0.093 | 0.031 | 0.911 | 0.838-0.991 |
